# Hydrolysis of Glycosidic Flavonoids during the Preparation of Danggui Buxue Tang: An Outcome of Moderate Boiling of Chinese Herbal Mixture

**DOI:** 10.1155/2014/608721

**Published:** 2014-03-16

**Authors:** Wendy Li Zhang, Jian-Ping Chen, Kelly Yin-Ching Lam, Janis Ya-Xian Zhan, Ping Yao, Tina Ting-Xia Dong, Karl Wah-Keung Tsim

**Affiliations:** Division of Life Science and Center for Chinese Medicine, The Hong Kong University of Science and Technology, Clear Water Bay Road, Hong Kong

## Abstract

Chemical change during boiling of herbal mixture is a puzzle. By using Danggui Buxue Tang (DBT), a herbal decoction that contains Astragali Radix (AR) and Angelicae Sinensis Radix (ASR), we developed a model in analyzing the hydrolysis of flavonoid glycosides during the boiling of herbal mixture in water. A proper preparation of DBT is of great benefit to the complete extraction of bioactive ingredients. Boiling of DBT in water increased the solubility of AR-derived astragaloside IV, calycosin, formononetin, calycosin-7-*O*-**β**-D-glucoside, and ononin in a time- and temperature-dependent manner: the amounts of these chemicals reached a peak at 2 h. The glycosidic resides of AR, calycosin-7-*O*-**β**-D-glucoside, and ononin could be hydrolyzed during the moderate boiling process to form calycosin and formononetin, respectively. The hydrolysis efficiency was strongly affected by pH, temperature, and amount of herbs. Interestingly, the preheated herbs were not able to show this hydrolytic activity. The current results supported the rationality of ancient preparation of DBT in boiling water by moderate heat.

## 1. Introduction

Herbal decoction, as a water extractive, is a common form of preparation in traditional Chinese medicines (TCMs). This water preparation, recorded in the earliest medicinal book “*52 Bing Fang (Prescriptions for Fifty-two Diseases)*”, has been used in China for more than 3,000 years. Among the 113 herbal formulae in “*Shanghan Lun*” by* Zhang Zhongjing* in* Han* dynasty, 95 of them were prepared as a form of decoction [1]. In preparing a TCM decoction, the boiling procedure in water had been demonstrated to be an efficient extracting method of active ingredients from the herbs [2, 3], which could efficiently extract the chemicals through multiple factors including temperature, solvent, and time. However, the chemical change during this boiling of herbal mixture is largely unknown, which impedes the understanding of our ancient wisdom of herbal preparation.

Among thousands of herbal decoction, Danggui Buxue Tang (DBT) is a simple combination of two herbs, which is commonly used as a health food supplement for women aliments. DBT was first described in* Neiwaishang Bianhuo Lun* by* Li Dongyuan* (AD 1247) in China.* Li *described the DBT formula that should include 10* qian* of Astragali Radix (AR), roots of* Astragalus membranaceus* (Fisch.) Bunge or* Astragalus membranaceus* (Fisch.) Bungevar.* mongholicus *(Bunge) P. K. Hsiao, and two* qian* of Angelicae Sinensis Radix (ASR), roots of* Angelica sinensis* (Oliv.) Diels. This formula should be boiled in two bowls of water over moderate heat until the final volume is reduced by half [3, 4]. Women with menopausal symptoms were prescribed to drink DBT daily to enrich their “*Qi*” and nourish their “*Blood*” [3]. Indeed, recent pharmacological results indicated that DBT had the ability to promote hematopoietic function, to stimulate cardiovascular circulation, to strengthen immune response, to increase insulin sensitivity, to prevent osteoporosis, and to act as antioxidants [5–10].

The AR-derived flavonoids, including formononetin, calycosin, ononin, and calycosin-7-*O-*β**-D-glucoside, were shown to play important roles in the functions of DBT, for example, osteogenic effect, estrogenic effect, and erythropoietic effect [11–13]. Our previous studies showed that the amount of chemicals derived from AR, soluble in DBT, was higher than the water extract derived from single herb of AR, and it is interesting that the amounts of formononetin and calycosin were dependent on the boiling conditions [7]. Moreover, the erythropoietic function of DBT in inducing hypoxia-inducible factor-1*α* (HIF-1*α*) was mediated by the AR-flavonoids, and interestingly the aglycone flavonoids showed better efficiency than that of the glycone partners [13]. By using DBT as an example here, we aimed to reveal the solubility and the possible chemical change of AR-derived astragaloside IV, calycosin, formononetin, calycosin-7-*O-*β**-D-glucoside, and ononin in the herbal decoction during boiling process. This study aimed to develop a model system in analyzing the dynamic change of chemicals during boiling of herbal mixture.

## 2. Materials and Methods

### 2.1. Plant Materials and Reagents

Three-year-old* Astragalus membranaceus* var.* mongholicus* roots from Shanxi province [14] and two-year-old* Angelica sinensis* roots from Minxian in Gansu province [15] were collected. These areas are known to produce the best quality of AR and ASR [4], respectively. The authentication of plant materials was identified morphologically by Dr. Tina Dong at Hong Kong University of Science and Technology (HKUST). Their corresponding voucher specimens as forms of whole plants, voucher number 11-08-4 for AR and voucher number 10-11-1 for ASR, were deposited in the Center for Chinese Medicine at the Hong Kong University of Science and Technology. Astragaloside IV was purchased from National Institute for the Control of Pharmaceutical and Biological Products (Beijing, China). Calycosin, formononetin, ononin, and calycosin-7-O-**β**-D-glucoside were purchased from Weikeqi Biotechnology Co. (Sichuan, China). The purities of these chemical markers, confirmed by high-performance liquid chromatography (HPLC), were higher than 98.0%. Analytical- and HPLC-grade reagents were purchased from Merck (Darmstadt, Germany).

### 2.2. Sample Preparation of DBT

Thirty gram of sliced AR and six gram of sliced ASR were mixed (the ratio is 5 : 1) and then boiled in 288 mL (w : v = 1 : 8) of water. To determine the accumulated amounts of AR-derived chemicals during boiling process, the temperature was controlled to rise 5°C every 10 min; after boiling for 30 min, the temperature was controlled to rise 10°C every 10 min until it reached 100°C. Every 10 min, 0.5 mL solution was withdrawn from the decoction before it reached 100°C. At 100°C, 0.5 mL solution was withdrawn every half an hour. The collected decoction was centrifuged at 16,000 ×g for 10 min at 4°C, and the supernatant was collected and stored at −20°C for LC/MS/MS analysis. To compare the amounts of AR-derived chemicals in DBT decoctions with different boiling process, two DBT decoctions were prepared, one was boiled using moderate heating (as described above), the other was boiled with high heating directly (the herbs were added onto boiling water directly for 4.5 h). After boiling, sample solution was withdrawn and centrifuged at 16,000 ×g for 10 min at 4°C, and the supernatant was collected and stored at −20°C for LC/MS/MS analysis.

### 2.3. Sample Preparation of Hydrolytic Analysis

To determine the effect of ASR or AR on the hydrolysis of flavonoid glycosides, a defined amount of calycosin-7-O-**β**-D-glucoside and ononin was added to the powder form of ASR or AR solution with different concentration. The AR powder was used as a blank for background calculation, since the four tested flavonoids were only existing in AR. The heated ASR and AR powders were done by heating at 100°C for 30 min in an oven. After 4 h, 0.5 mL of the solution was withdrawn from each sample, the solution was centrifuged at 16,000 ×g for 10 min at 4°C, and the supernatant was collected and stored at −20°C for further LC/MS/MS analysis. In the testing of pH and temperature, similar procedure was done. For pH values 3 and 4, glycine-HCl buffer was used, and for pH values 5 and 6 sodium phosphate buffer was used, while sodium phosphate buffer was used for pH value 7, and the pH value was adjusted by HCl and/or NaOH.

### 2.4. Chemical Analysis by RRLC-QQQ-MS/MS

An Agilent 1200 series system (Agilent, Waldbronn, Germany), which was equipped with a degasser, a binary pump, an autosampler, and a thermo-stated column compartment, was used for the analysis. The chromatographic condition was as described previously [16]. For the MS/MS analysis, an Agilent QQQ-MS/MS (6410A) equipped with an ESI ion source was used for all analyses. The marker chemicals in DBT were quantified as fully described previously [16].

### 2.5. Cell Culture

Human mammary epithelial carcinoma MCF-7 cells and human embryonic kidney fibroblast (HEK) 293T cells were obtained from American Type Culture Collection (ATCC, Manassas, VA). MCF-7 cells were grown in modified Eagle's medium (MEM), supplemented with 10% fetal bovine serum, 2 mM L-glutamine, 0.1 mM nonessential amino acids, 1 mM pyruvate, 100 units/mL penicillin, and 100 units/mL streptomycin in a humidified CO_2_ (5%) incubator at 37°C. HEK-293T cells were cultured in Dulbecco's modified Eagle's medium (DMEM), supplemented with 10% fetal bovine serum, 100 units/mL penicillin, and 100 units/mL streptomycin in a humidified CO_2_ (5%) incubator at 37°C. All culture reagents were purchased from Invitrogen Technologies (Carlsbad, CA).

### 2.6. Estrogenic and Erythropoietic Assay

Three repeats of estrogen responsive elements (ERE: 5′-GGT CAC AGT GAC C-3′) were synthesized and then subcloned into a luciferase-reporter vector called pTAL-Luc (BD Biosciences Clontech, San Jose, CA) to form pERE-Luc. This reporter was stably transfected to MCF-7 cells [17]. After transfection with the luciferase reporter gene construct and drug treatment for 48 h, cultures were then collected by lysis buffer containing 0.2% Triton X-100, 1 mM dithiothreitol, and 100 mM potassium phosphate buffer, pH = 7.8, and which was then subjected to luciferase and protein assays. Luciferase assay was performed by a commercial kit (Tropoc Inc., Bedford, MA). The readings corresponding to luciferase activities were quantified by FLUOstar Optima (BMG Labtech), where luciferase activities were normalized per mg of protein in each sample.

Six repeats of hypoxia responsive elements (HRE: 5′-TCG AGG CCC TAC GTG CTG TCT CAC ACA GCC TGT CTG ACG-3′) were synthesized, concatemerized, and then cloned in tandem (head-to-tail orientation) into pBI-GL vectors (BD Biosciences Clontech) that had a downstream reporter of firefly luciferase gene [4]. This vector was named as pHRE-Luc [18]. Cultured HEK293T cells were transiently transfected with pHRE-Luc by the calcium phosphate precipitation method [19]. The transfection efficiency was over 80%, as determined by another control plasmid that is having a**β**-galactosidase, under a cytomegalovirus enhancer promoter. After transfection and drug treatment for 48 h, cell lysate was collected, and luciferase activity was determined as in estrogenic assay in MCF-7 cells.

### 2.7. Statistical Analysis and Other Assays

Protein concentrations were measured routinely by Bradford's method with a kit from Bio-Rad Laboratories (Hercules, CA). Statistical tests were done by using one-way analysis of variance. Data were expressed as Mean ± SD, where *n* = 3. Statistically significant changes were classified as significant (∗), where *P* < 0.05 and highly significant (∗∗), where *P* < 0.01.

## 3. Results 

### 3.1. Quantification of the Change of Chemicals during Boiling Process of DBT

**Figure 1:**
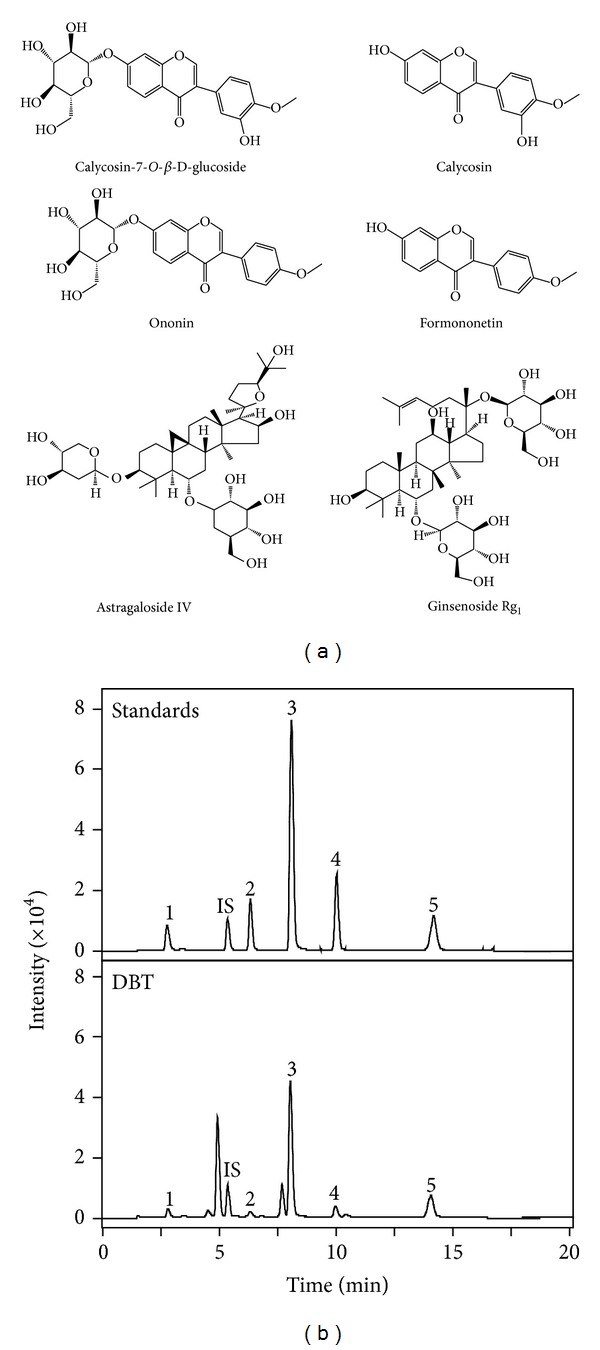
*Typical RRLC-QQQ-MS/MS chromatograms of marker chemicals in DBT.* (a) The chemical structures of calycosin-7-*O*-**β**-D-glucoside, calycosin, ononin, formononetin, astragaloside IV, and ginsenoside Rg1 (internal standard) were shown. (b) The chromatographic method is described in Section 2. The identifications of calycosin-7-O-**β**-D-glucoside (1), ononin (2), calycosin (3), astragaloside IV (4), formononetin (5), and ginsenoside Rg1 (internal standard, IS) were made by a MS detector. Concentrations of the standards were 0.2 *μ*g/mL, and 0.5 mL of the DBT decoction was used. Representative chromatograms are shown, *n* = 3.

**Figure 2:**
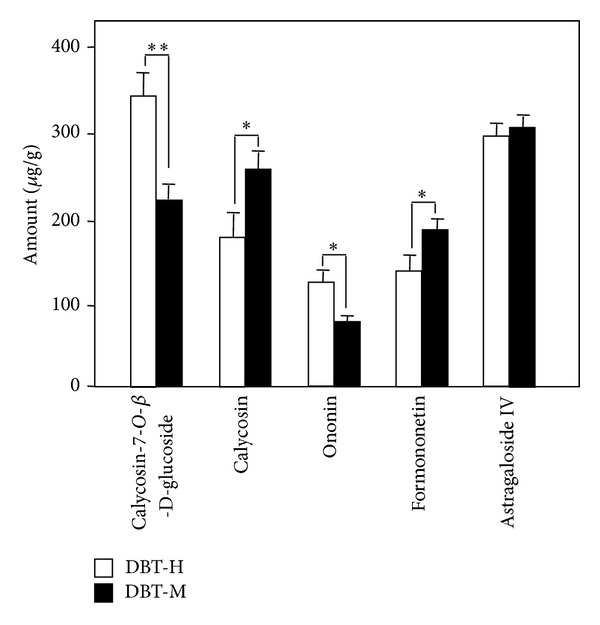
*The amounts of AR-derived chemicals in DBT under different boiling process.* Two DBT decoctions were prepared, one was boiled using moderate heating (DBT-M), the other one was boiled with high heating directly (DBT-H). Detailed procedure was described in Section 2. The amounts of calycosin-7-O-**β**-D-glucoside, calycosin, ononin, formononetin and astragaloside IV were determined. The values are expressed in *μ*g/g of the dry material and are in Mean ± SD, where *n* = 4, each with triplicate samples. The amount of the same compound was compared in these two decoctions, *P < 0.5, **P < 0.01.

DBT was prepared under two different boiling processes, one was boiled using moderate heat (denoted as DBT-M), while the other one was boiled under strong heating directly (denoted as DBT-H). After boiling, the two decoctions (DBT-M or DBT-H) were subjected to chemical analysis. The amounts of AR-derived astragaloside IV, calycosin, formononetin, calycosin-7-*O-*β**-D-glucoside, and ononin were determined. The chemical structure of these markers (Figure 1(a)) and their characteristics in the MS/MS (Table 1) were revealed. The typical MS/MS chromatograms of the eight markers, as well as the included internal standard, in the herbal extracts were shown in Figure 1(b). The method validation was done as fully described previously [16]. Our results showed that the two DBTs showed significant variation in chemical composition (Figure 2). Higher amount of glucoside nonconjugated flavonoids, for example, calycosin and formononetin was revealed in DBT-M, while the glucoside conjugated flavonoids, for example, calycosin-7-*O-*β**-D-glucoside and ononin, were significantly higher in DBT-H. The amount of astragaloside IV was unchanged. In addition, the amounts of ASR-derived chemicals, including ferulic acid and Z-ligustilide, during the two boiling conditions in DBT, were identical, that is, ferulic acid (1330.05 ± 107.4 *μ*g/1 g) and Z-ligustilide (137.57 ± 12.5 *μ*g/1 g), mean ± SD, *n* = 4 [16]. Thus, the heating process of DBT should play role in the final chemical composition of the decoction, in particular the glucoside flavonoids.

**Figure 3:**
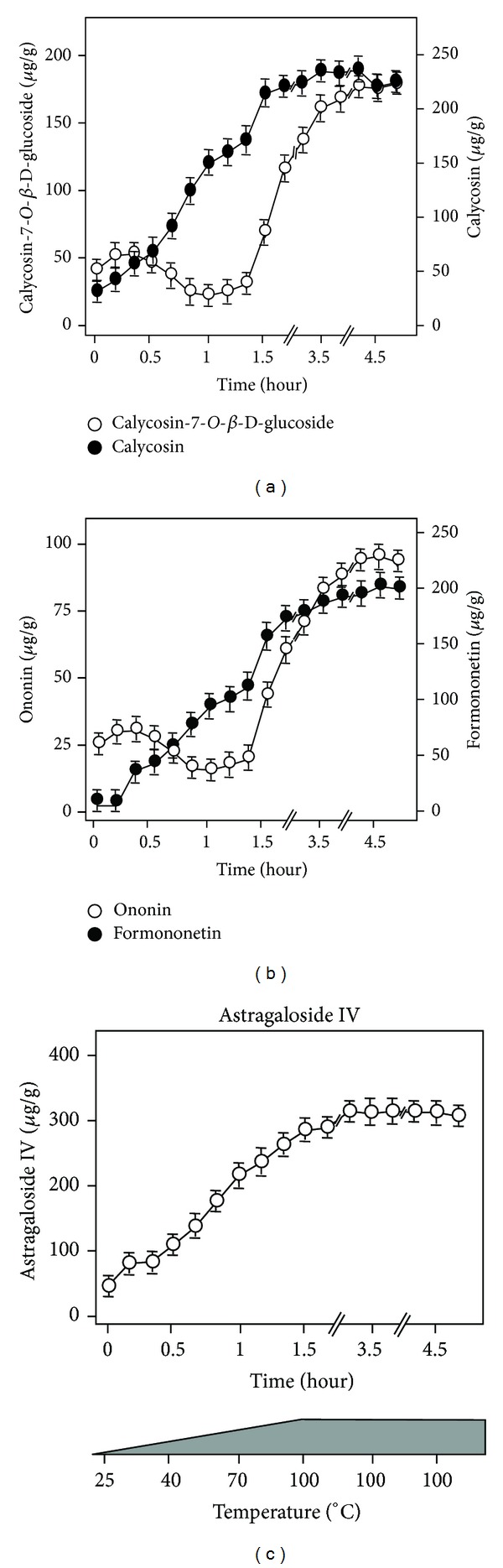
*The accumulated amounts of AR-derived chemicals during boiling process.* The DBT was prepared as described in Section 2. Every 10 min, 0.5 mL of the preparing decoction was taken out. The temperature was recorded at the time of taking aliquot (see (c) bottom panel). After ~1.5 h, the boiling temperature reached 100°C, and the time interval of withdrawing sample was 30 min. In all aliquots, the amounts of calycosin-7-O-**β**-D-glucoside, calycosin, ononin, formononetin, and astragaloside IV were determined by RRLC-QQQ-MS/MS. The values are expressed in *μ*g/g of the dry material and are in Mean ± SD, where *n* = 4, each with triplicate samples.

The amounts of AR-derived astragaloside IV, calycosin, formononetin, calycosin-7-*O-*β**-D-glucoside and ononin were determined during the entire boiling process at a moderate heating rate of totally 5 h. At each time point, an aliquot of decoction was obtained: the temperature and amount of targeted chemicals were determined. The accumulated amounts of these chemicals were changed during the boiling process (Figure 3).

The AR-derived flavonoid glycosides, calycosin-7-*O-*β**-D-glucoside, and ononin were increased in parallel to the time of boiling in the very beginning. However, the amounts of both calycosin-7-*O-*β**-D-glucoside and ononin were decreased when the boiling temperature reached 40°C at ~0.5 h of boiling: the lowest solubility was achieved at about 70°C at 1 h. Beyond that, the amounts of these two chemicals were steadily increased to a maximum after boiling 2 h at a temperature of 100°C (Figure 3). The AR-derived flavonoid aglycones, calycosin, and formononetin showed similar profile of changes during the boiling process. The rate of increase was faster at 1 to 1.5 h of boiling (i.e., from 70°C to 100°C). The amount of these two flavonoid aglycones increased in a time- and temperature-dependent manner and reached their maximum after boiling for 2 h at 100°C (Figure 3). However, calycosin and formononetin did not show a drop of solubility at ~0.5 h of boiling. In contrast, the rate of increase was slightly higher at that time period. Similar changes of solubility were observed in astragaloside IV (Figure 3).

### 3.2. Effect of Herbs on Hydrolysis of Calycosin-7-O-*β*-D-glucoside and Ononin

**Figure 4:**
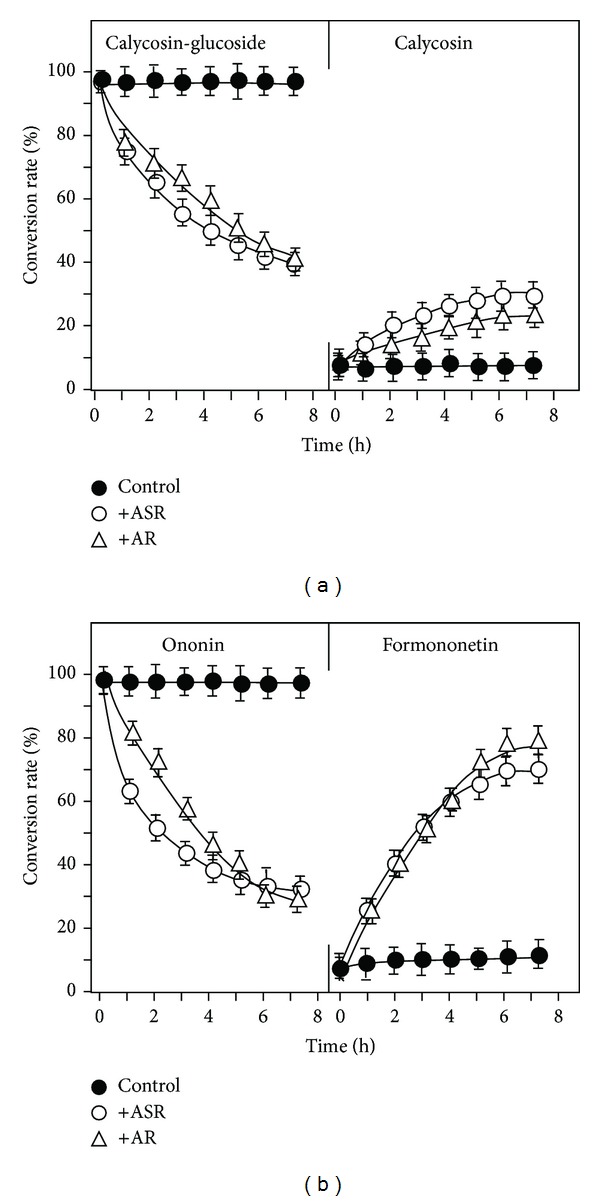
*A time-dependent hydrolysis of flavonoid glycosides in the present of herbal powder.* Under 25°C, 50 *μ*g of calycosin-7-O-**β**-D-glucoside (a), or 50 *μ*g ononin (b), was included to the water (50 mL) suspended with powders (1 g) of ASR or AR. Every hour, 0.5 mL of the solution was taken out. The amounts of calycosin-7-O-**β**-D-glucoside, calycosin, ononin, formononetin, and astragaloside IV were determined by RRLC-QQQ-MS/MS as described in Figure 1. The percentage of hydrolysis rate was determined by the amount of flavonoid or flavonoid glycoside (in molar) determined to that original applied flavonoid glycoside (in molar). Control does not have herbal powder. The values are in Mean ± SD, where *n* = 4, each with triplicate samples.

The amount of calycosin-7-*O*-**β**-D-glucoside and ononin decreased during boiling at the temperatures of 40°C to 70°C that suggested that a possible chemical change could happen, that is, a hydrolysis of calycosin-7-*O*-**β**-D-glucoside to calycosin and from ononin (formononetin 7-O-glucoside) to formononetin. Here, we hypothesized that the flavonoid glycosides, deriving from AR, could be converted to their aglycones by hydrolysis during the heating process. In order to test this hypothesis, the stabilities of calycosin, formononetin, ononin, and calycosin-7-*O-*β**-D-glucoside were tested here. The results showed that the flavonoid glycosides were very stable in water for over 6 h without any significant chemical change (Figures 4(a) and 4(b)). Similarly, the aglycones, calycosin, and formononetin were also highly stable in water (data not shown). A robust chemical conversion was revealed when the flavonoid glycosides were mixed together with a solution of ASR or AR at room temperature. After 4 h of incubation with water suspended with powders of ASR or AR, about 50% of calycosin-7-*O-*β**-D -glucoside disappeared. In contrast, an increased amount of calycosin (Figure 4(a)) suggested the hydrolysis of glycoside, that is, a conversion of calycosin-7-O-**β**-D -glucoside to calycosin. The hydrolysis of flavonoid glycoside was also determined for the case of ononin to formononetin. The decreased of ononin amount was more robust during the incubation: about 50% of decrease was revealed in an inclusion of ASR solution after ~2 h (Figure 4(b)). An increase of formononetin was revealed here, which reached a peak at about 6 h of incubation. In both cases of hydrolysis, ASR showed much better effect than that of AR, which suggested that the hydrolytic ability (or factor) could be more abundant in ASR.

**Figure 5:**
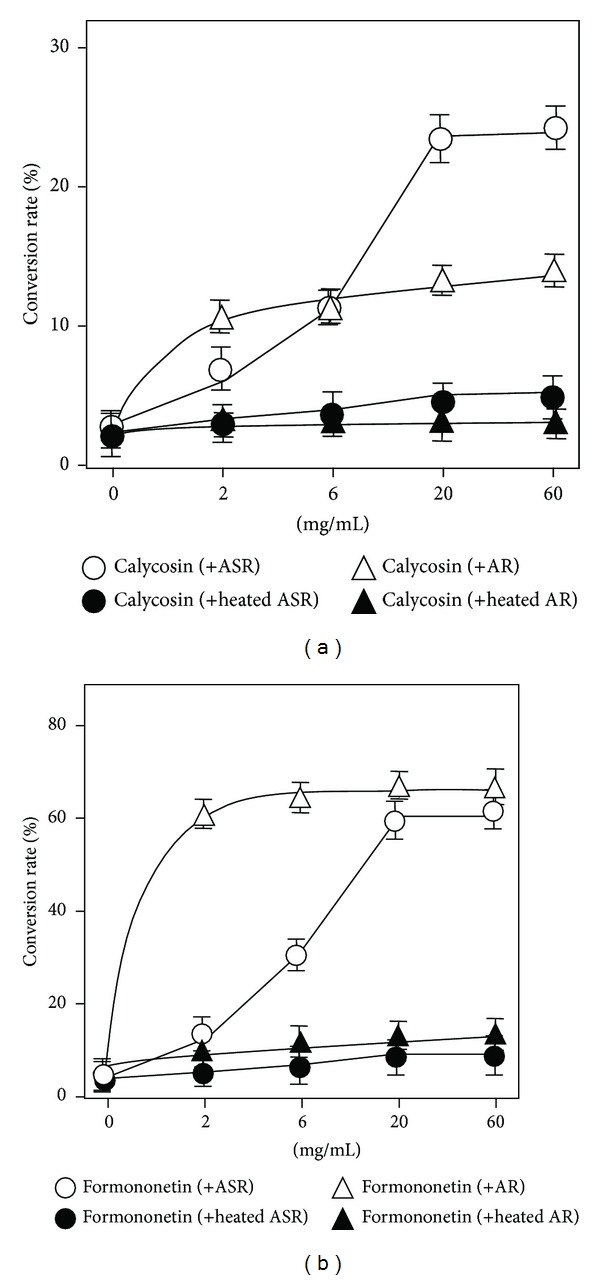
*The herb-induced hydrolysis is in a dose-dependent manner.* Under 25°C at pH 5.0, 50 *μ*g of calycosin-7-O-**β**-D-glucoside, or 50 *μ*g ononin, was included to the water (50 mL) suspended with different amount of ASR or AR powders for 4 h. Both crude and heated (30 min at 100°C) herbal powders were used here. The amounts of calycosin-7-O-**β**-D-glucoside, calycosin, ononin, and formononetin were determined by RRLC-QQQ-MS/MS. The percentage of hydrolysis rate was determined by the amount of flavonoid or flavonoid glycoside (in molar) determined to that original applied flavonoid glycoside (in molar). (a) Conversion from calycosin-7-O-**β**-D-glucoside to calycosin. (b) Conversion efficiency of ononin to formononetin. The values are in Mean ± SD, where *n* = 4, each with triplicate samples.

Since the chemical conversion happened with a company of ASR or AR, a dose responsible curve was determined. The results showed that the hydrolysis of two flavonoid glycosides was increased by ASR or AR in a dose-dependent manner (Figures 5(a) and 5(b)). In all cases, 20 mg/mL of herbal powder showed the maximal hydrolytic ability. In addition, the hydrolysis induced by ASR or AR was more robust in the case of ononin to formononetin, that is, a maximum conversion of over 60%. Almost no hydrolysis was revealed when the preheated ASR (i.e., denatured ASR), or preheated AR, solution was mixed together with the flavonoid glycosides (Figures 5(a) and 5(b)). This result suggested that the hydrolytic factor should be heat sensitive. The hydrolytic ability of AR seemed to be in a faster rate than that of ASR.

**Figure 6:**
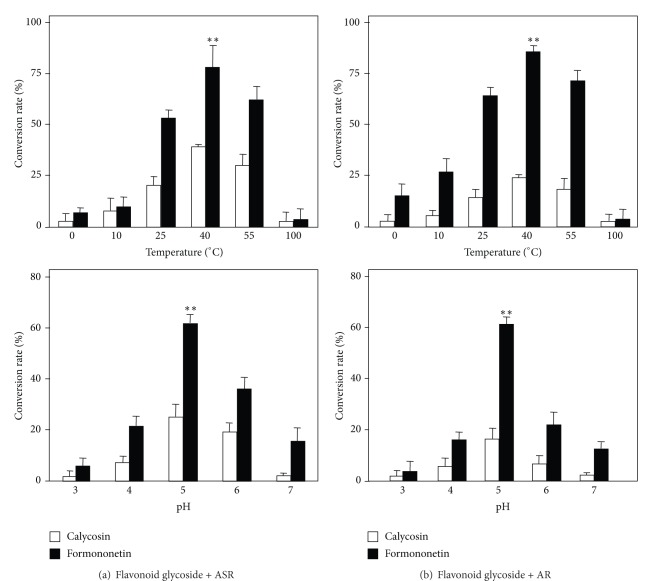
*Effect of temperature and pH on the hydrolysis of flavonoid glycosides.* Under different temperature at pH 5.0, or under different pH at 25°C, 50 *μ*g of calycosin-7-O-**β**-D-glucoside or ononin was included to the water (50 mL) suspended with powders of ASR (a) or AR (b) (both at 1 g) for 4 h. The amounts of calycosin-7-O-**β**-D-glucoside, calycosin, ononin, and formononetin were determined by RRLC-QQQ-MS/MS. The percentage of hydrolysis rate was determined by the amount of flavonoid or flavonoid glycoside (in molar) determined to that original applied flavonoid glycoside (in molar). The values are in Mean ± SD, where *n* = 4, each with triplicate samples. **P < 0.01.

Different parameters could affect the chemical hydrolysis here. The best temperature in ASR- or AR-induced hydrolysis was at 40°C (Figures 6(a) and 6(b) upper panel): this optimal temperature was true for both calycosin-7-O-**β**-D-glucoside and ononin. At the extreme low and high temperatures, both ASR and AR could not able to trigger this hydrolysis. On the same time, the best pH for hydrolysis, triggered by ASR or AR, was at pH 5 (Figures 6(a) and 6(b) lower panel). Indeed, this pH was the natural pH of water mixed with herbs without any adjustment. In all cases, the hydrolysis of ononin to formononetin was more robust than that of calycosin-7-O-**β**-D-glucoside to calycosin.

### 3.3. Biological Properties of DBT Prepared under Different Boiling Process

**Figure 7:**
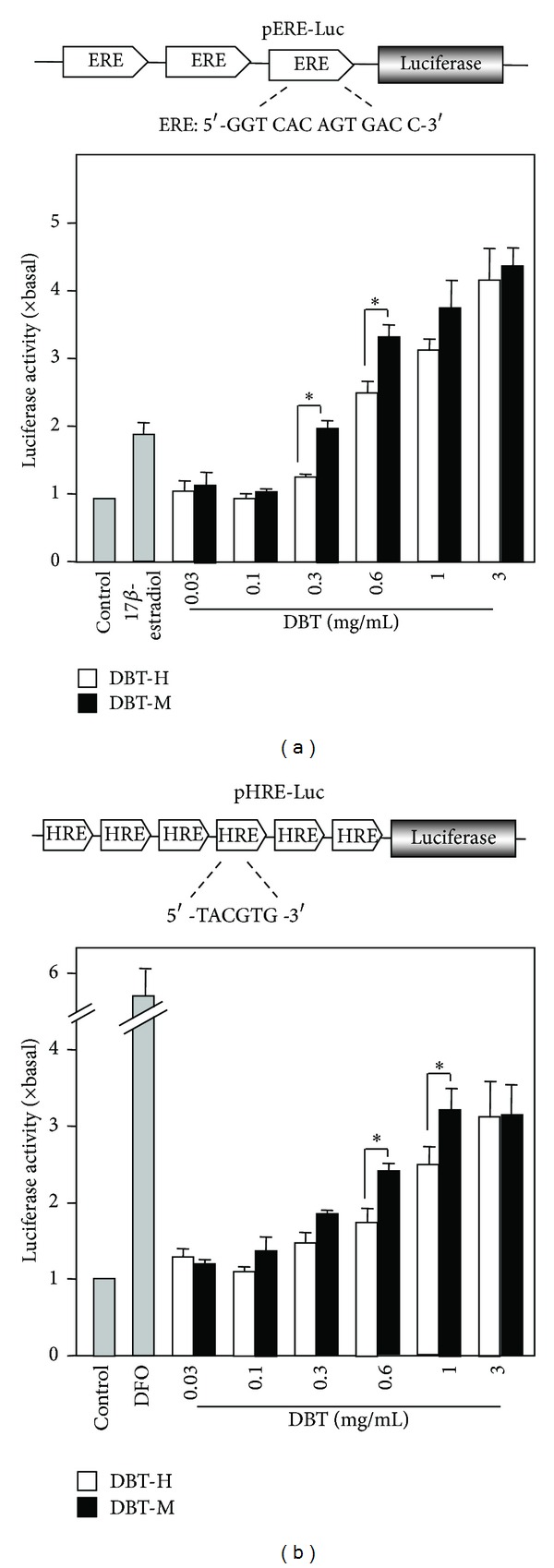
*DBT stimulates transcriptional activity of estrogen responsive element and the HRE-mediated transcriptional activity.* (a) Three repeats of estrogen responsive elements (ERE: 5′-GGT CAC AGT GAC C-3′) were subcloned into a luciferase-reporter vector called pERE-Luc (upper panel). This reporter was stably transfected to MCF-7 cells, which were treated with DBT extracts at different dose for 48 h (lower panel). 17-**β**-Estradiol (100 nM) was used as a positive control. (b) Six repeats of hypoxia responsive elements (HRE: 5′-TCG AGG CCC TAC GTG CTG TCT CAC ACA GCC TGT CTG ACG-3′) were subcloned in an expression vector and it was named as pHRE-Luc (upper panel). DBT extracts at different concentrations were applied to the pHRE-Luc-transfected HEK293T cells for 48 h; the promoter-driven luciferase (pHRE-Luc) activity was determined. DFO (50 *μ*M) was used as positive controls. Values are expressed as the ratio to the basal reading where the control (untreated culture) is equal to 1 and are in Mean ± SD, where *n* = 4, each with triplicate samples. *P < 0.05.

According to our previous results, DBT possessed estrogenic activity on MCF-7 cells and stimulation of the transcriptional activity of hypoxia response element (HRE) in HEK293T cells, which is a specific hematopoietic growth factor for the production of erythropoietin (EPO). The biological functions of the two DBT decoctions, DBT-M, and DBT-H were also compared here. For the estrogenic activity, MCF-7 cells stably transfected with pERE-Luc (Figure 7(a) upper panel) were employed. After the treatment of DBT for 48 h, the cultures were collected for luciferase assay. The expression of luciferase was markedly induced by DBT in a dose-dependent manner (Figure 7(a)). The maximal induction of the luciferase activity, induced by the two DBT extracts (DBT-M and DBT-H), was almost the same, which was ~4.5-fold at 3 mg/mL. However, the DBT-M extract showed stronger activity at lower concentrations, that is, at 0.3 or 0.6 mg/mL. 17-**β**-estradiol at 100 nM was used as a positive control here, which caused ~2-fold increase in the promoter activity.

The transcriptional activity of HRE induced by DBT was investigated. The response of the pHRE-Luc in transfected HEK 293T cells was validated; the cultures were exposed to hypoxia, serving as a positive control. The authentication of pHRE-Luc was confirmed by its activation in exposure to desferrioxamine (DFO), which was frequently used to mimic the effect of hypoxia [20]. DBT was applied onto pHRE-Luc transfected fibroblasts. The application of the extracts increased the luciferase activity in a dose-dependent manner, and the maximal induction was over ~3-fold of increase as compared to the control (Figure 7(b)). Although the maximal induction of the two DBT was almost the same at the highest concentration, significant difference was observed under lower concentration, for example, when the concentration was 0.6 mg/mL and 1 mg/mL. Under these concentrations, DBT-M showed stronger activity than the DBT-H.

## 4. Discussion

Making a herbal decoction by water is the most common form of preparation [9, 21], and indeed this method has been used for thousands of years in China. Using boiling water as a solvent for herbal extraction could due to the below reasons: (i) boiling water could be a perfect solvent for high solubility of herbal ingredients; (ii) absorption of water decoction could be easier in the gut; (iii) water decoction could be easily modified to other forms, for example, powder and pill, according to the needs of different patients. As shown here, the boiling process of herbal mixture could be a complex process, and a lot of unknown chemical changes could happen during the preparation [9, 22]: this change might affect the extraction efficiency of active ingredients as well as the functions of herbal decoction. In case of DBT as described here, the solubility of AR-derived flavonoids and astragalosides were increased in a time- and temperature-dependent manner during the boiling process at moderate heating. The amounts of these chemicals reached their maximum after boiling 2 h at 100°C: this result was consistent with our previous study that 2 hours of boiling at moderated heat were chosen for the standard preparation of DBT [3]. In parallel, the pH of a herbal mixture in water was about 5, that is, the optimal pH for the hydrolysis.

The increased amount of calycosin and formononetin, generated partly from calycosin-7-O-**β**-D-glucoside and ononin, enhanced the functions triggered by DBT. Our study here showed that DBT-M possessed better biological functions in stimulating the estrogenic and erythropoietic effects than that of DBT-H. Although the aforementioned four flavonoids could induce the expression of erythropoietin in cultured kidney cells and stimulate the estrogenic activity in cultured mammalian breast cancer cells, the induction magnitude of aglycones is better than their corresponding glycosides [12, 13]. Besides, the transporting rate of calycosin-7-O-**β**-D-glucoside and ononin in cultured Caco-2 cells were considered as low permeability [23, 24], while calycosin and formononetin were showing much better permeability [23]. Better absorption of active ingredients in the gut is important, because the intake of DBT is orally. Therefore, the hydrolysis of calycosin-7-O-**β**-D-glucoside and ononin could generate more active ingredients that could be easily absorbed: the final outcome is to ensure better biological functions of DBT.

According to TCM formulation, herbal decoction can be divided into different categories by their major functions, for example, exterior syndromes, purgative, and tonic functions. The preparation for herbal mixture having different therapeutic functions should be varied. According to “*Bencao Gangmu”* by* Li Shenzhen,* the herbal formulae for treating exterior syndrome and purgative function should be boiled over strong heating, while the herbal formulae for tonic function should be boiled over moderate heat. DBT is considered as a classical herbal formula for nourishing* Qi* and enriching* Blood*; therefore it is strongly recommended to be boiled with moderate heat. Under the optimal temperature (~40°C) during DBT preparation, both calycosin-7-O-**β**-D-glucoside and ononin could undergo hydrolysis in forming calycosin and formononetin, respectively. The glycoside hydrolysis required the present of two herbs, ASR or AR, contained within DBT, and the hydrolysis was optimized in a specific temperature and pH. Thus, the final outcome of this moderate boiling of DBT was to provide a condition that the hydrolysis could happen, and thus the final decoction was optimized. Here, we provided different lines of evidence to support the ancient preparation method for a tonic herbal formula. We also suggested that one should try to maintain the temperature at ~40°C for some time as to achieve the best decoction.

The hydrolysis of flavonoid glycosides in DBT could be affected by different factors, that is, amount of herbs, temperature, and pH. The heated herbs, both ASR and AR, were not able to show this hydrolytic ability. Here, we hypothesize that the hydrolytic ability could be triggered by an enzyme that is existing in both ASR and AR. This notion is fully supported by our results. First, the hydrolysis was highly sensitive to temperature and pH value. Second, a dose response curve and reaching saturation could be achieved in the herb-induced hydrolysis. Lastly, the hydrolysis of glycosidic bonds was reported to be triggered by enzymes [25]. However, the exact reason of the chemical conversion should be investigated further. This hydrolytic activity does not apply to all herbal powders; for example,* Ginseng* Radix et Rhizoma is only able to induce the conversion of calycosin-glucoside to calycosin, which cannot induce the conversion of ononin to formononetin, while* Clematidis Radix et Rhizoma* cannot do that at all (data not shown).

## 5. Conclusion

In conclusions, the chemical change during the boiling process of DBT was first studied here by using RRLC-QQQ-MS/MS method. The solubilities of the flavonoids were markedly increased in a time- and temperature-dependent manner. Flavonoid glycoside within the decoction was hydrolyzed to aglycone, which was affected by pH, temperature, and amount of herbs. Our current results supported the rationality of ancient preparation of DBT in boiling water by moderate heat.

## Figures and Tables

**Table 1:** Mass spectra properties of marker chemicals.

Chemical	Formula	Calculated mass [M]	Precursor ion [M − H]^a^	Fragmentor energy^b^	Collision energy^c^	Production ion^d^	Retention time (min)^e^
Formononetin	C_16_H_12_O_4_	268.1	267.1	150	17	252	13.966
					29	223	
Astragaloside IV	C_41_H_68_O_14_	784.9	829.5^f^	190	5	829.5	9.873
					25	783.2	
Calycosin	C_16_H_12_O_5_	284.1	283.1	100	13	268	7.894
					29	211	
Ononin	C_22_H_22_O_9_	430.4	465.1	80	5	267.1	6.257
					23	252.1	
Calycosin-7-*O*-*β*-D-glucoside	C_22_H_22_O_10_	446.1	481	120	5	283.1	2.749
					27	268.1	
Ginsenoside Rg_1_	C_42_H_72_O_24_	800.5	799.5	250	5	799.5	5.293
					21	637.3	

^a^The detected chemicals had the greatest responses under the negative mode: the [M − H]^−^ was used as the precursor ion.

^b^The fragmentor energy was optimized to have the greatest ionize efficiency.

^c^The collision energy was optimized to have the greatest product ion intensity, which was the key factor in the MRM mode.

^d^Two product ions were used for the MRM analysis. The upper one was used for quantitative analysis and the lower one was for qualitative analysis, which could guarantee the precision of analytes.

^e^The retention time was determined by 3 different individual analyses (*n* = 3).

^f^The precursor ion of astragaloside IV was [M + HCOOH − H]^−^ under the negative mode.

## Abbreviations

DBT: Danggui Buxue Tang
AR: Astragali Radix
ASR: Angelicae Sinensis Radix
TCM: Traditional Chinese Medicine.
